# Four Requisites in Development

**Published:** 1895-12

**Authors:** M. F. Ault

**Affiliations:** Kokomo, Ind.


### THE DENTAL REGISTER.

Vol. XLIX.] DECEMBER, 1895. [No. 12.

### COMMUNICATIONS.

### Four Requisites in Development.

BY M. F. AULT, M.D., D.D.S., KOKOMO, IND.

*Mr. President and Members of the Tri State Dental Association:*

It has not been my privilege to be among the incorporators of a Dental Society, but I am grateful to the pioneers who introduced the idea of associate work and thereby have kept alive the spirit of our vocation and enforced the respectful consideration of society. My hope is that we may have sufficient courage to be loyal to the truth as it is in dentistry, and by our devotion subdue the empiric and convince those who have been led into the jungles by the wily promises of the impostor. These remarks bring us face to face with the fact that the god which presides over dental destiny has triumphed in but few hearts, that the process of true education has yet wonders to perform and that the conflict of the future will be as the conflict of the past—a struggle between truth and falsehood, intelligence and ignorance, honor and depravity.

To prove that the conflict of the future will be as the conflict of the past, let us review briefly our present situation and compare it with a practical ideal. The elements which enter into our experience and influence our opinions and consequent actions are as follows:

1. The Novice or embryo.
2. The College.
3. The Profession.
4. The Professional.
5. The People.

Now, the inharmonious relation of the elements has made bitter the experience of the past, and the substitution of an harmonious relation will be the occasion for controversy and upheavals, as well as to inspire us with the sweetness of ultimate success.

1. The novice of the past, with his crude notions, knocked for admission. He moved on the low plane of a money basis. Sought the most for the least service and, with few exceptions, passed an uneasy life and finally closed his office to be hurried through the Eternal gate clothed in that mantel which the caprice and selfishness of the world is prone to weave.

The novice of the future will be of the same character except the modification which we expect through the direct and reflex influence of true education. We want him to know that when he knocks at our door he is asking admission into a sacred dominion. That he is helpless, on account of his ignorance. That he is dust and soon to mingle with the elements. That he is in distress and needs some one to show him the true way.

2. The colleges of the past and present while maintaining an apparently innocent front have worked too much on a revenue basis—have too often prostituted themselves for money and thereby augmented the ranks of those whose motive is impure, whose action is demoralizing and whose touch is fatal.

I anticipate that the college of the future will not disappoint us in the fulfillment of its mission. The future college will say to the aspirant we know you seek the truth and it shall be opened unto you. We know you are timid, but confidence, courage and ability shall be yours. We know you know nothing about the exacting and inflexible laws of dental practice, but the inspiration within these walls will quicken you into new life and pave the way for a prosperous career. The future college will address the applicant in this manner: Do you think you possess the natural ability to become a desirable member of our profession? Do you love learning to the extent that we may expect an earnest effort to obtain knowledge? Is your ideal sufficiently elevated to make your desire the dignity of a cultured character? Can you think for yourself? Have you the concentration that enables one to surmount great difficulties? And do you possess patience and humility and realize your own littleness sufficiently to be *teachable*?

The future college will say to its faculty: A teacher's preparation must be determined by the nature of the subjects to be taught and the end to be accomplished by the recipient of the instruction. If you are able to teach, you realize that the true thought of a subject, is God's thought and your task is to make the pupil's thought the same. You will see in the pupil an immature judgment, inaccurate observation, uncertain memory, perverted imagination, dishonest intentions, and feeble will, and as a teacher you must correct the misconceptions by the educational process. Do you know what is meant by chaos? that is the pupil's mental condition and your mission as a faculty is to illuminate the mind, train the hand and lead your students back to God. The dental college of the future will say to all the students and teachers: I am the way and my way is aggressive unto development and purification. Take up your cross and follow me.

3. Among the elements which enter into our experience one is faultless and that one is the profession. I refer to the profession not as so many individuals but as a list of entities which constitute the grand total of discovered and undiscovered dental knowledge. It contains the truth of the past and future. The truths of a profession are of a divine origin—they constitute the relentless laws which are not penned by human hands—laws which are abiding as the eternal throne, because they are not characterized by human frailty and lobbied through a weak and vascilating legislature. Who is it that is not aware of the law of inspection in the human eye, the law of touch in the human mouth, the law of pain in the human teeth, and of speech in the human tongue? What man does not quail when he undertakes to reproduce nature and introduce his handiwork to the delicate sensibility which prevades the oral organs? These laws were enacted on that day when it was said. "Let us make man in our own image." This is the imperial edict before which all must bow and in it we see the safety of our profession. These statutes may appear merciless but their truths touch a cold heart and transform it into a center of sympathy, touch a benighted mind and reveal the knowledge of divine power, touch the eye before which flit ignoble visions and open it to the light which shines from a pure throne, touch the ear in which has lodged the notes of beastly composition and make it sensitive to the pure song, and righteous instruction.

4. Professional is an appellation which we apply to those already in practice and without making the past professional the subject of comment we will say that the practitioner of the future will correspond in character to the college which sends him forth —taking as our premises the law that like begets like, or that the face and character grows into harmony with the life that we live. I am aware that we are disposed to talk much about blood, title and pedigree, but I feel at this juncture that environment, association or personal contact is entitled to more consideration, and that the institution which gives the learners the best environment and closest personal attention will send men forth who are able to perform the miracle of teaching the truth and can honorably say that they have faithfully attended the required course of instruction and passed satisfactorily examinations upon all branches incident to a dental curriculum.

5. The people are a heterogeneous mass which will be elevated in proportion to the reflex from the college. In the description of the people we can use many terms without doing violence—such as fake, mountebank, deceit, suspicious, unsympathetic, selfish, ignorant, also consistency, self-denial, charity, faith, fidelity, and love.

Notwithstanding the fact that our vocabulary description of meanness may far surpass our list descriptive of goodness, I have great faith in human nature, treacherous as it may sometimes seem. Let no man deceive himself by thinking that honor is obsolete and as he passes through the wavering assemblage he must suffer the swallowing of both soul and body. Much as we may fancy our profession to be demoralized through the efforts of the impostor or the alleged demands of the multitudes we cannot afford to have the future exegesis read: He denied his identity—he lost his individuality—he ceased to be positive—he laid down his instrument one fatal day and sought his abode to die and this is his epitaph: "He was a panderer—He was treacherous to his calling, championed no theory which looked to advancement and leaves the record of a misguided life—He surrendered —had the capacity for a great life—knew it, but did it not."

To recapitulate: The novice, college, profession, professional and people constitute the elements of dental experience and foregoing comment places the college in the very responsible position of determining the future status of dentistry. A port, if properly fortified, prohibits the landing of an enemy, but if the walls are low and the garrison small and poorly-equipped plunder and devastation are sure to follow. This is the relation of the college to the domain occupied by our brethren. Every one who entered the French pavilion at the exposition was certainly impressed with "The Guardian Spirit of the Secrets of the Tomb," and each as he looked upon the powerful arm which embraced the urn of ashes had his own revery and made his own application. After placing myself in full sympathy with the sentiments of the sculptor I thought how truly also is this a symbol of educational institutions. The guardian spirit is the faculty and the urn the receptacle of God's mysteries, but in this the spirit must lift the lid and invite the inquirer to view the beauties within.

Now, so far as I have observed, it is the belief of not only the pessimist but of optimist that our ports need strengthening and to do this let us turn our attention. Three essentials must be considered before the college can meet the expectations of the times:

(*a.*) Specific Scientific Instruction.

(*b.*) Specific Manual Instruction.

(*c.*) Specific Soul Instruction.

We wish to place particular emphasis to the first, because we are wandering in the scientific department and the pupil is failing to see the relation of much of our scientific instruction to his development.

In the second or manual department the difficulty is in keeping each section profitably employed and the third or soul instruction usually ignored.

[*To be Continued.*]

